# The effect of physical activity on cytokine levels in adults living with type 1 diabetes—a preliminary study

**DOI:** 10.14814/phy2.15985

**Published:** 2024-04-19

**Authors:** Małgorzata Kowal, Renata Woźniacka, Anna Ścisłowska‐Czarnecka, Joanna Homa, Wojciech Głodzik

**Affiliations:** ^1^ Department of Anthropology, Faculty of Physical Education and Sport Bronislaw Czech University of Physical Education Kraków Poland; ^2^ Department at Cosmetology, Faculty of Motor Rehabilitation Bronislaw Czech University of Physical Education in Krakow Krakow Poland; ^3^ Department of Evolutionary Immunology Institute of Zoology and Biomedical Research, Jagiellonian University Krakow Poland; ^4^ Sanatio Medical Center Krakow Poland

**Keywords:** cardiovascular disease (CVD), cytokines, fatness, physical activity, type 1 diabetes mellitus (T1DM)

## Abstract

The aim of this study is to investigate whether physical activity and the level of body fat are factors reducing the level of pro‐inflammatory cytokines in people with T1DM. Twenty‐five men (27.8 ± 9.4 years old; 178.9 ± 6.9 cm; 80.6 ± 12 kg) and 18 women (28.1 ± 12.5 years old; 162.4 ± 5.5; 63.1 ± 9.9 kg) were divided into four groups based on body fat percentage and level of physical activity (AN—active people with normal body fat; IAN—inactive people with normal body fat; AO—active people with excessive body fat, IAO—inactive people with excessive body fat). The level of cytokines in the blood serum was assessed. The level of IL‐8 was higher (measurable) in inactive men, regardless of adiposity degree and in women, only in the inactive group with normal body fat. IL‐6 was found only in active men with excessive adiposity. In conclusion, the findings from this study allow to indicate that moderate level of physical activity may contribute to a reduction in the development of systemic low‐grade inflammation in patients with T1DM, and thus, may reduce the risk of CVD.

## 1 INTRODUCTION

Type 1 diabetes mellitus (T1DM) is a disorder characterized by destruction of pancreatic beta cells, which ultimately leads to absolute insulin deficiency. Most often, this disease results from autoimmune‐mediated destruction of beta cells, although a small minority of cases result from idiopathic destruction or failure of beta cells (Maahs et al., 2010). Early symptoms of these disease are related to hyperglycemia and include polyuria, polydipsia as well as weight loss, even with increased appetite. This is an outcome of insulin deficiency (Cai et al., 2021). In recent years, the incidence of T1DM has increased significantly due to changes in people's behavior and lifestyle (Rosengren & Dikaiou, 2023). It turns out that selected environmental factors may activate autoimmune mechanisms in people with a genetic predisposition to develop type 1 diabetes, which may contribute to the loss of pancreatic beta cells. Such factors include infections during the perinatal period, viral infections (e.g., cytomegalovirus), too early replacement of natural feeding with cow's milk in children, as well as toxins contained in food (e.g., dietary nitrosamines) (Zimmet et al., 2001).

Hence, this chronic disease has become a key public health problem due to increased morbidity and mortality associated with it (Chiesa & Marcovecchio, 2021). The increasing prevalence of T1DM in younger people is successively leading to higher rates of long‐term complications such as retinal and kidney disease, neuropathy, and cardiovascular disease (CVD) (Chiesa & Marcovecchio, 2021). This results in the prolonged medical care of these people which contributes to a significant increase in the cost and utilization of medical care services (Htay et al., 2019).

The increased risk of CVD in patients with T1DM (Teoh et al., 2021) is caused by chronic hyperglycemia the result of which can potentially be, among others, oxidative stress, vascular inflammation, monocyte adhesion, arterial wall thickening, and endothelial dysfunction (Vergés, 2020). As a result, CVD is the major cause of mortality in T1DM and contributes to 30%–44% of all deaths among affected individuals (Teoh et al., 2021). In recent studies, a downward trend has been shown in both CVD and mortality over the past few decades. However, the risk of cardiovascular death still remains still 4.2 times higher in people with T1DM compared to non‐diabetic controls (Colom et al., 2021; Teoh et al., 2021). The additional factors increasing the risk of CVD in people with T1DM include hypertension, dyslipidemia or insulin resistance (Wu et al., 2021; Rosa, Oliver, et al., 2011), physical inactivity, poor diet as well as smoking (Chiesa & Marcovecchio, 2021), and recently, excessive body mass (Polsky & Ellis, 2015). To date, people with T1DM have been perceived as being of healthy body mass. However, in recent years, the problem of excess body mass has been observed among individuals with T1DM and, according to estimates, this issue currently affects 60% of them. This is the effect of a steep increase in the occurrence of overweightness and obesity in the global population (Rawshani et al., 2018). Overweightness and obesity in people with T1DM are accompanied by insulin resistance, the consequence of which is hyperinsulinemia, dyslipidemia, and chronic inflammation, and thus, cancer. Apart from CVD, cancer has become the main cause of premature death among individuals with T1DM (Fellinger et al., 2019; Rawshani et al., 2018).

The heightened risk of CVD in patients with T1DM seems to be the result of systemic inflammation caused by immune system cells, but also by dysfunctional adipocytes or endothelial cells, cytokines, and oxidative stress (Rosa, Oliver, et al., 2011). The effect of increased pro‐inflammatory cytokine levels, such as interferon‐γ (IFN‐γ), IL‐1β, IL‐6, and TNF‐α, may result in the accumulation and subsequent activation of macrophages in the endothelium of blood vessels. Activated macrophages, producing cytokines and chemokines, can cause the formation and rupture of atherosclerotic plaques, thus, contributing to an elevated risk of CVD (Amin et al., 2020; Haybar et al., 2019).

Both of these phenomena are caused by disturbances in the levels of insulin, glucose, cortisol, lipoproteins, or oxidative enzymes (Kawai et al., 2021; Rosa, Oliver, et al., 2011).

One of the factors reducing the risk of CVD is exercise. Codella et al. (2017) found that regular physical activity improves glucose tolerance in patients with T1DM and T2DM by further improving insulin sensitivity and beta cell efficiency (Codella et al., 2017). Regularly performing exercise also reduces systemic inflammation and restores proper balance between anti‐ and pro‐inflammatory cytokines (Rosa, Heydari, et al., 2011). In the physical activity further decreases the influence of risk factors such as hypertension and dyslipidemia in the case of CVDs. At the same time, it causes beneficial changes in body composition. This is done through the reduction of adipose tissue is the body. Exercise is also known to improve the well‐being of patients with T1DM (Scott et al., 2019). In individuals with T1DM, regularly undertaking moderate physical activity is recommended, such as brisk walking or biking. It should be performed for a minimum of 150 min accumulated over at least 3 non‐consecutive days of the week, but also at higher levels (≥240 min/week) (Plotnikoff et al., 2006). For low intensity exercise, 30 min or more is recommended per day (Wu et al., 2021). Unfortunately, despite the many benefits of regular physical activity, people with T1DM are less active than their healthy peers (Plotnikoff et al., 2006). This is mostly due to a lack of adequate knowledge regarding exercise management and hypoglycemic episodes (Wu et al., 2021).

The aim of this study is to investigate whether physical activity and the level of body fat are factors that contribute to reduction in the level of pro‐inflammatory cytokines among T1DM patients.

## 2 MATERIALS AND METHODS

### 2.1 Participants

The study participants comprised 25 men (age: 27.8 ± 9.4 years; body height: 178.9 ± 6.9 cm; and body mass: 80.6 ± 12 kg) and 18 women (age: 28.1 ± 12.5 years; body height: 162.4 ± 5.5 cm; and body mass: 63.1 ± 9.9 kg). All the participants having T1DM had been diagnosed with the disease at least 1 year prior to commencing the study (average disease duration was 13.4 ± 8.81 years in men and 13.44 ± 9.11 in women) (Table 1). The subjects were characterized by absence of other chronic diseases.

**TABLE 1:** Characteristics of study participants among adults living with type 1 diabetes.

	Male	Female
*N*	25	18
Age (years)	27.84 ± 9.44	28.15 ± 12.55
Body weight (kg)	80.61 ± 11.95	63.07 ± 9.86
Body height (cm)	178.86 ± 6.91	162.39 ± 5.72
BMI (kg/m^2^)	25.29 ± 3.65	23.87 ± 2.97
WHR	0.83 ± 0.06	0.76 ± 0.05
WHtR	0.48 ± 0.06	0.46 ± 0.04
Fat mass (%)	20.55 ± 5.76	29.71 ± 4.99
Weekly physical activity (MET/min)	1668.5 ± 1228.45	1181.94 ± 1056.66
DM1 duration (years)	13.40 ± 8.81	13.44 ± 9.11

Inclusion criteria:

- age between 20 and 40 years;
- having controlled T1DM;
- absence of other chronic diseases;
- consent to participate in the research.

Exclusion criteria:

- age above 20 and below 40 years;
- having uncontrolled T1DM;
- other chronic diseases, including other types of diabetes;
- no consent to participate in research.

### 2.2 Procedures

#### 2.2.1 Anthropometric measurements

Anthropometric measurements were obtained according to the Martin‐Saller technique (Martin & Saller, 1957). Body height was measured to the nearest 0.1 cm using an anthropometer (Switzerland), and body mass to the nearest 0.1 kg with the Tanita TBF‐300A scale (Japan). Waist and hips circumferences were measured using a non‐stretchable tape with an accuracy of 5 mm, the measuring instrument placed directly on the skin at the level of the iliac crest (for the waist) and at the maximum extension of the buttocks (for the hip). Skinfold thickness was measured using the Holtain skinfold caliper (Holtain Ltd., United Kingdom) with dial graduation of 0·2 mm.

Four skinfolds were measured:

- triceps, over the midpoint of the triceps muscle between the olecranon process and acromion;
- subscapular, just below the scapula at a 45°‐angle to the lateral side of the body;
- suprailiac, with the natural angle of the iliac crest at the anterior axillary line; and
- abdominal, diagonally, midway between the umbilicus and right anterior superior iliac spine.

All measurements of the adipose folds were taken on the right side of the body. In order to estimate overall body fatness as well as the distribution of adipose tissue, the sum of three (triceps, abdominal, and suprailiac) and four skinfolds was calculated.

Additionally, based on the abovementioned measurements, the following indicators were calculated: body mass index (BMI) (body mass [kg]/body height^2^ [m]), waist‐to‐hip ratio (WHR) (waist circumference [mm]/hip circumference [mm]), waist‐to‐height ratio (WHtR) (waist circumference [mm]/body height [mm]), and body fat percentage (BF%) (BF% = 1.20 × BMI + 0.23 × age – 10.8 × gender −5.4; where men = 1, women = 0) (Daurenberg et al., 1991). The 85 percentile value was used as a cutoff points, which for women was 30.8% and for men 20.1% of body mass (McCarthy et al., 2006).

#### 2.2.2 Level of physical activity

To assess the physical activity level of the respondents, the shortened version of the International Physical Activity Questionnaire (IPAQ) (Biernat et al., 2007) was used. Based on the data collected in the questionnaire, the physical activity of the participants was determined in units of MET‐min/week. Those who obtained a result below 600 MET‐min/week were qualified into the group of people performing an insufficient amount of physical activity, while those who obtained a result above 600 MET‐min/week were qualified into the group demonstrating a sufficient level of physical activity (Bergier et al., 2012).

#### 2.2.3 Level of cytokines in the blood serum

The level of cytokines in the blood serum of the participants was determined via flow cytometry using Flex Sets (CBA), according to manufacturer guidelines (Cytometric Bead Array, BD Biosciences, USA). In the study, the Humane Inflammation Kit (BD Biosciences, USA) was applied, which allows simultaneous determination for the level of six cytokines: interleukin 1β (IL‐1β), interleukin 6 (IL‐6), interleukin 8 (IL‐8), interleukin 10 (IL‐10), interleukin 12p70 (IL‐12p70), and tumor necrosis factor (TNF‐α). Data analyses and determination of cytokine concentrations were performed in Microsoft Excel using standard curves, which were made on the basis of consecutive dilutions of the standard. The lowest detection level was 4.88 (pg/mL).

#### 2.2.4 Level of HbA_1c_ (glycated hemoglobin) and ACR (albumin/creatinine ratio)

During testing, a blood sample was taken from each participant and sent to a diagnostic laboratory gauging the level of HbA_1c_ and ACR.

All the participants were divided into four groups based on BF% and level of physical activity:

- AN—active people (result above 600 MET‐min/week) with normal body fat (below 30.8% for women and 20.1% for men);

Numbers: *N* = 8 women; *N* = 10 men;

- IAN—inactive people (result below 600 MET‐min/week) with normal body fat (below 30.8% for women and 20.1% for men);

Numbers: *N* = 2 women; *N* = 3 men;

- AO—active people (result above 600 MET‐min/week) with excessive body fat (above 30.8% for women and 20.1% for men);

Numbers: *N* = 3 women; *N* = 10 men;

- IAO—inactive people (result below 600 MET‐min/week) with excessive body fat (above 30.8% for women and 20.1% for men);

Numbers: *N* = 5 women; *N* = 2 men.

All measurements were performed by experienced researchers during one appointment. The researchers were blinded to subject group allocation. This study was of cross‐sectional design. The participants were, in detail, informed about the study protocol and signed written informed consent forms for participation in the study. All procedures were carried out in accordance with the 1964 Declaration of Helsinki and its later amendments. The Bioethics Committee at the Regional Medical Association in Kraków (No. 196/KBL/012/2019) approved the study.

### 2.3 Statistical analysis

Demographic information and the results are presented as group means ± standard deviations (SD). ANOVA was applied to assess the significance of statistical differences between all sex‐distinguished groups, followed by Tukey's post hoc test. The Student's *t*‐test was applied to compare the significance of differences between the adiposity of men and women within the selected groups according to low and sufficient level of physical activity (AN vs. IAN and AO vs. IAO). The significance level was set at *p* < 0.05. The analysis was performed using Statistica StatSoft 13 software.

## 3 RESULTS

Based on the obtained results, it was concluded that in the group of men characterized by a sufficient level of physical activity, regardless of body fat degree, the difference in the level of physical activity expressed in MET/min/week in relation to inactive men was statistically significant (*p* < 0.05). In the group of women, the level of physical activity was statistically significantly different only among women with excessive adiposity.

There were no differences in age or mean values of the analyzed somatic features and anthropological indices between groups of men with a normal level of body fat and characterized by different levels of physical activity, which allowed these groups to be considered homogeneous. In groups of men with excessive adiposity and different levels of physical activity, it was noted that the differences in the mean values of the analyzed features were also not statistically significant. However, analysis of the remaining variables showed that men from IAO group, compared to men from AO group, were statistically significantly heavier and were also characterized by a statistically significantly increased risk of central adiposity (Table 2). In the group of women with normal and increased levels of adiposity, between groups of women varying in the level of physical activity, no differences were found in age or mean values of the analyzed somatic features and anthropological indices. This allowed all groups to be considered comparable (Table 3). Analyzing the distribution of adipose tissue, based on the thickness of skinfolds, in both men and women, regardless of adiposity, no differences were found between groups characterized by different levels of physical activity. Therefore, it was considered that both the group of women and the group of men were homogeneous groups (Tables 4 and 5).

**TABLE 2:** Descriptive data of the male sample and anthropometric, biochemical, and inflammatory parameters of them.

Male	Active individuals, normal body fat (AN) Mean ± SD	Inactive individuals, normal body fat (IAN) Mean ± SD	Statistical differences between active and inactive individuals with normal body fat	Active individuals with excessive fatness (AO) Mean ± SD	Inactive individuals with excessive fatness (IAO) Mean ± SD	Statistical differences between active and inactive individuals with excessive fatness
Age (years)	23.8 ± 4.7^a^	20.6 ± 1.5^a^	*p* = 0.28	31.0 ± 9.7^ab^	42.9 ± 14.3^b^	*p* = 0.16
Body weight (kg)	74.7 ± 7.6^a^	73.3 ± 2.4^a^	*p* = 0.77	85.5 ± 13.5^ab^	96.7 ± 0.42^b^	*p* = 0.28
Body height (cm)	180.1 ± 5.9	184.5 ± 3.0	*p* = 0.25	176.8 ± 8.2	174.7 ± 4.6	*p* = 0.75
BMI (kg/m^2^)	23.03 ± 2.1^a^	21.6 ± 0.6^a^	*p* = 0.27	27.4 ± 2.4^b^	31.8 ± 2.0^c^	***p* = 0.04**
WHR	0.78 ± 0.03^a^	0.78 ± 0.04^ab^	*p* = 0.86	0.85 ± 0.04^b^	0.96 ± 0.05^c^	***p* = 0.009**
WHtR	0.44 ± 0.03^a^	0.42 ± 0.02^a^	*p* = 0.36	0.51 ± 0.03^b^	0.62 ± 0.04^c^	***p* = 0.002**
Fat mass (%)	16.9 ± 2.8^a^	14.4 ± 0.7^a^	*p* = 0.17	23.8 ± 3.1^b^	31.8 ± 5.7^b^	***p* = 0.01**
Weekly physical activity (MET/min)	2206.2 ± 1365.8^a^	400.0 ± 229.1^b^	***p* = 0.049**	1813.7 ± 867.4^a^	156.2 ± 221.0^b^	***p* = 0.03**
DM1 duration (years)	11.1 ± 3.6^a^	9.0 ± 1.0^a^	*p* = 0.35	16.9 ± 12.1^a^	14.0 ± 14.0^a^	*p* = 0.30
HbA_1c_ (%)	7.4 ± 1.0^a^	7.3 ± 1.9^a^	*p* = 0.87	7.1 ± 0.7^a^	7.3 ± 1.0^a^	*p* = 0.7
IFCC HbA_1c_ (mmol/mol)	57.3 ± 10.7^a^	55.7 ± 20.6^a^	*p* = 0.85	53.8 ± 7.3^a^	56.0 ± 11.3^a^	*p* = 0.72
ACR (mg/L)	0.59 ± 0.62^a^	0.25 ± 0.07^a^	*p* = 0.49	0.36 ± 0.3^a^	0.95 ± 0.7^a^	*p* = 0.13
IL – 6 (pg/mL)**	–	–	–	83.9 ± 36.7	–	–
IL‐8 (pg/mL)**	–	57.19 ± 49.7	–	–	7.09	–

^a,b^ Significance of differences between all four distinguished groups.

** In the groups where data were not provided, the level of IL was not detectable. Significance of differences was not determined for these groups either.

**TABLE 3:** Descriptive data of the female sample and anthropometric, biochemical, and inflammatory parameters of them.

Female	Active individuals, normal body fat (AN) Mean ± SD	Inactive individuals, normal body fat (IAN) Mean ± SD	Statistical differences between active and inactive individuals with normal body fat	Active individuals with excessive fatness (AO) Mean ± SD	Inactive individuals with excessive fatness (IAO) Mean ± SD	Statistical differences between active and inactive individuals with excessive fatness
Age (years)	20.5 ± 2.5^a^	21.6 ± 0.4^ab^	*p* = 0.56	38.1 ± 15.3^ab^	36.2 ± 15.0^b^	*p* = 0.87
Body weight (kg)	60.2 ± 10.7	58.0 ± 7.0	*p* = 0.80	68.3 ± 9.1^ab^	65.4 ± 10.0^b^	*p* = 0.69
Body height (cm)	164.1 ± 6.6	162.5 ± 6.4	*p* = 0.77	160.8 ± 4.6	160.5 ± 6.1	*p* = 0.93
BMI (kg/m^2^)	22.2 ± 2.3^a^	21.9 ± 0.9^b^	*p* = 0.88	26.4 ± 3.0^b^	25.3 ± 2.1^c^	*p* = 0.61
WHR	0.74 ± 0.04	0.73 ± 0.03	*p* = 0.64	0.77 ± 0.05^b^	0.80 ± 0.1^c^	*p* = 0.54
WHtR	0.44 ± 0.03^a^	0.43 ± 0.02^ab^	*p* = 0.75	0.50 ± 0.04^b^	0.50 ± 0.06^c^	*p* = 0.95
Fat mass (%)	26.0 ± 2.7^a^	25.9 ± 1.0^a^	*p* = 0.93	35.0 ± 3.2^b^	33.3 ± 1.9^b^	*p* = 0.44
Weekly physical activity (MET/min)	1668.7 ± 1193.1	125.0 ± 0.0	*p* = 0.12	1420.0 ± 615.3^a^	191.7 ± 269.6^b^	***p* = 0.02**
DM1 duration (years)	8.2 ± 3.9^a^	13.5 ± 3.5^ab^	*p* = 0.12	23.0 ± 11.9^a^	11.3 ± 4.5^a^	*p* = 0.16
HbA_1c_ (%)	7.5 ± 0.7^a^	6,6 ± 0.2^a^	*p* = 0.16	7.1 ± 1.1^a^	7.3 ± 1.3^a^	*p* = 0.47
IFCC HbA_1c_ (mmol/mol)	58.5 ± 7.9^a^	49.0 ± 2.8^a^	***p* = 0.02**	63.2 ± 12.9^a^	56.0 ± 14.2^a^	*p* = 0.51
ACR (mg/L)	2.12 ± 1.0^a^	0.3 ± 0.2^a^	*p* = 0.53	0.57 ± 0.5^a^	0.23 ± 0.1^a^	*p* = 0.39
IL – 6 (pg/mL)**	–	–	–	–	–	–
IL‐8 (pg/mL)**	–	99.4	–	–	–	–

^a,b^ Significance of differences between all four distinguished groups.

** In the groups where data were not provided, the level of IL was not detectable. Significance of differences was not determined for these groups either.

**TABLE 4:** The median (Me) and mean values and standard deviation (*x* ± SD) of skinfolds thickness, and their sum in male samples.

	Active individuals, normal body fat (AN)		Inactive individuals, normal body fat (IAN)		Statistical differences between AN and IAN	Active individuals with excessive fatness (AO)		Inactive individuals with excessive fatness (IAO)		
	Me	*x* ± SD	Me	*x* ± SD		Me	*x* ± SD	Me	*x* ± SD	Statistical differences between AO and IAO
Triceps (mm)	9.75	10.75 ± 5.12	10.0	10.33 ± 3.51	*p* = 0.89	12.5	14.60 ± 7.97	16.25	16.25 ± 9.54	*p* = 0.80
Abdominal (mm)	17.0	19.20 ± 7.61^a^	15.0	16.67 ± 5.69^ab^	*p* = 0.61	28.5	28.40 ± 10.11^bc^	40.0	40.00 ± 21.21^c^	*p* = 0.23
Subscapular (mm)	13.5	14.50 ± 3.50^a^	11.0	10.70 ± 0.52^a^	*p* = 0.10	21.0	22.90 ± 8.82^b^	26.0	33.00 ± 7.07^b^	*p* = 0.16
Suprailliac (mm)	20.5	20.50 ± 6.54^a^	15.0	16.33 ± 4.16^a^	*p* = 0.33	31.0	30.30 ± 10.76^b^	35.0	35.00 ± 5.66^b^	*p* = 0.57
Sum of 3 folds (mm)	42.0	44.45 ± 12.68^a^	33.0	37.70 ± 8.93^a^	*p* = 0.41	65.5	65.90 ± 23.35b	89.25	89.25 ± 37.83^b^	*p* = 0.26
Sum of 4 folds (mm)	61.5	64.95 ± 18.65^a^	48.0	54.03 ± 13.04^a^	*p* = 0.37	97.0	96.20 ± 32.20^b^	124.25	124.25 ± 43.49^b^	*p* = 0.30

^a,b^ Significance of differences between all four distinguished groups.

**TABLE 5:** The median (Me) and mean values and standard deviation (*x* ± SD) of skinfolds thickness, and their sum in female samples.

	Active individuals, normal body fat (AN)		Inactive individuals, normal body fat (IAN)		Statistical differences between AN and IAN	Active individuals with excessive fatness (AO)		Inactive individuals with excessive fatness (IAO)		Statistical differences between AO and IAO
	Me	*x* ± SD	Me	*x* ± SD		Me	*x* ± SD	Me	*x* ± SD	
Triceps (mm)	14.5	15.50 ± 4.75	19.0	19.00 ± 1.41	0.35	15.0	17.00 ± 4.35	21.0	21.80 ± 7.29	0.34
Abdominal (mm)	23.0	23.37 ± 6.54	22.0	22.00 ± 1.41	0.78	22.0	27.00 ± 9.54	32.0	30.00 ± 3.08	0.52
Subscapular (mm)	17.5	19.62 ± 6.46	17.0	17.00 ± 1.41	0.60	30.0	27.00 ± 13.74	26.0	28.00 ± 4.85	0.88
Suprailiac (mm)	17.5	20.69 ± 7.25	18.5	18.50 ± 7.78	0.71	21.0	26.00 ± 16.09	28.0	27.40 ± 2.70	0.85
Sum of 3 folds (mm)	55.5	58.5 ± 14.36^a^	58.0	58.00 ± 1.41^ab^	0.96	65.0	71.00 ± 25.53^ab^	77.0	79.80 ± 12.77^b^	0.53
Sum of 4 folds (mm)	74.0	79.19 ± 19.99^a^	76.5	76.5 ± 6.36^ab^	0.86	86.0	97.00 ± 41.60^ab^	100.0	107.20 ± 14.27^b^	0.61

^a,b^ Significance of differences between all four distinguished groups.

Taking the percentage of HbA_1c_ and ACR (albumin/creatinine ratio) determined in the blood serum into account, no statistically significant differences were found between the compared groups of women and men. At the same time, the HbA1c percentage did not exceed 7.5%, which may indicate a similar level of diabetes control in each group. This allows to assess the impact of physical activity and adiposity on cytokine levels, excluding the impact of diabetes itself.

By analyzing the levels of interleukin 1β (IL‐1β), interleukin 6 (IL‐6), interleukin 8 (IL‐8), interleukin 10 (IL‐10), interleukin 12p70 (IL‐12p70), and tumor necrosis factor (TNF‐α), increased levels of only IL‐8 and IL‐6 were found in selected groups. It was shown that the level of IL‐8 was higher (detectable) in inactive men, regardless of adiposity degree (IAN and IAO). In women, the level of this interleukin was detectable only among inactive women with normal adiposity. A significant level of IL‐6 was found in active men with excessive adiposity. This cytokine was not measured for women in any of the groups.

## 4 DISCUSSION

Based on the obtained results, it was found that the examined people with T1DM did not differ in the level of HbA_1c_ or ACR. Nonetheless, both women and men with excessive adiposity were characterized by a longer average disease duration compared to their peers with normal adiposity levels. An increased level of IL‐6 was found only in men declaring a sufficient level of physical activity and characterized by excessive adipose tissue. However, elevated levels of IL‐8 were found in inactive men from both adiposity groups and in inactive women with its normal levels.

It is well‐known that an excess of body fat can lead to hyperlipidemia and the related stimulation of macrophages in adipose tissue, resulting in increased production of pro‐inflammatory cytokines (Rosa, Heydari, et al., 2011). On the other hand, it seems that diabetes, per se, and associated hyperglycemic episodes may also contribute to elevated levels of pro‐inflammatory cytokines in the body (Rosa, Heydari, et al., 2011). It should be emphasized here that low‐grade systemic chronic inflammation plays a crucial role in the pathogenesis of not only cardiovascular, metabolic, and autoimmune diseases, but also of cancers and psychiatric disorders (Ferrucci & Fabbri, 2018; Marynowski et al., 2021; Silveira Rossi et al., 2022). One of the ways to lower the level of pro‐inflammatory cytokines circulating in the blood is physical activity (Karstoft & Pedersen, 2016; Nocon‐Bohusz & Noczyńska, 2016; Rosa, Oliver, et al., 2011). In the authors' research, beneficial effects of physical activity manifested in a low (undetectable) level of IL‐8 were found in men, regardless of body fat level, while in women, such effects were noted only in the group of females with normal body fat. A reverse trend was found for IL‐6. Elevated levels of this cytokine were observed only in men with excessive body fat, but declaring a sufficient level of physical activity. Therefore, the obtained results of the present study seem to be equivocal, which may be the result of the fact that the majority of researchers analyze changes in the level of cytokines during and shortly after exercise. Nevertheless, the long‐term effects of regular training on their level are rarely analyzed. It should also be added that for inflammation, the types of physical activity are also important (aerobic or resistance training). In research conducted in Belgium, it was shown that several weeks of resistance training increased the level of IL‐8, but only in the group of men (Forti et al., 2017). In the study carried out in Brazil, during which the participants were subjected to interval training, it was demonstrated that the change in IL‐8 level depended on training intensity. In the case of high‐intensity training, the level of IL‐8 increased during exercise, both in people with normal and excess body mass, and after 30 min, a decrease occurred in the level of this interleukin. In the case of moderate‐intensity training, such changes were observed in subjects with excess body mass. In individuals with normal body mass, the level of IL‐8 did not change during but decreased after training (Dorneles et al., 2016). Research in Tunisia using 12‐week interval training of moderate‐ or high‐intensity conducted on young women allowed to demonstrate a similar trend, but the positive result on adiponectin levels was already visible with moderate‐intensity exercise (Racil et al., 2013). Adiponectin is a fat‐derived hormone secreted into circulation. Adiponectin stimulates fatty acid oxidation in the skeletal muscles and inhibits glucose production in the liver, resulting in improved whole‐body energy homeostasis. Therefore, it seems that adiponectin may play an important role in the treatment of diabetes (Wang & Scherer, 2016). Adiponectin is also an anti‐inflammatory agent in various cell types and, in this way, also protects vasculature, the heart, lungs, and colon (Fang & Judd, 2018). In the present study, it was found that in the respondents' physical activity contributed to a reduction in IL‐8 levels. This may be related to the fact that in the individuals under study, the assessment of cytokine level did not take place immediately after completion of the exercises. The differences between the results of the authors' research and others known from literature on the subject may be the result of the fact that most of the research is performed on healthy individuals. Observations have shown, however, that in the case of subjects with the metabolic syndrome, physical activity may determine the level of cytokines in a different way (Trøseid et al., 2004). For example, research conducted in Norway on adults with metabolic syndrome allowed to indicate that physically active individuals exhibited lower levels of IL‐8 than those who were inactive. It should be emphasized here that in the case of this trial, the cytokine level was determined a few days after completing the training cycle, and the training cycle itself included endurance type exercises such as walking/jogging on treadmill, as well as strength training (Trøseid et al., 2004). Similar results were obtained in Canada. Research conducted at the basic level for adults and for adults with type 2 diabetes covers the effects of two types of a 12‐week training program: high‐intensity interval training (twice a week for 45 min), as well as continuous training of low to moderate intensity (twice a week lasting 1 h)—on the level of pro‐inflammatory cytokines. It was shown that regardless of the type of training, both in subjects with type 2 diabetes and in those from the control group, the levels of IL‐6 and IL‐8 were significantly reduced, and this effect was more visible in patients with type 2 diabetes (Garneau et al., 2023). The results of the trial seem to be consistent with those obtained in the present study, as it was demonstrated that in people declaring a sufficient level of physical activity, the level of IL‐8 was lower than in those from the physically inactive group. The results of the current study could have also been influenced by the fact that the examined persons had a stabilized glucose level (<120 mg/dL). It turns out that the way of controlling diabetes is of great importance in maintaining a high level of IL‐8. It has been shown that people with both type 1 and type 2 diabetes, compared to healthy people, are characterized by increased levels of IL‐8 in the blood serum. This is probably due to the fact that increased blood glucose levels promote the adhesion of monocytes to the vascular endothelium and the production of the abovementioned interleukin by these cells. Elevated levels of this interleukin therefore accelerate inflammation in people with diabetes and are also seen as a predictor of CVD (Shirzaiy et al., 2023; Zozuliñska et al., 1999). Type 1 or type 2 diabetics whose blood sugar levels have been stabilized demonstrated lower levels of IL‐8 compared to those with poorly controlled diabetes (Van Sickle et al., 2009). Due to the fact that all the studied people were under constant medical supervision and had well‐controlled diabetes, it can be assumed that the increased level of IL‐8 in the IAN and IAO groups was the result of the low physical activity performed by these participants. Based on the level of IL‐8, it is also worth noting that, as it seems, the level of cytokines was not related to the level of the subjects' adiposity, because the detectable level of this cytokine was found in the group of inactive people, regardless of degree of adiposity (Van Sickle et al., 2009). Similar results were obtained by researchers in Mexico. They conducted research on individuals with T1DM, without and with metabolic syndrome. What is important, people with metabolic syndrome were characterized by greater fat content than those from the reference group. It was found that subjects from either groups did not differ in the level of the analyzed cytokines (IL‐6, IL‐8, IL‐10) (Ferreira‐Hermosillo et al., 2015). Therefore, this may indicate that individuals from both groups had a similar state of inflammation. The differences in the level of cytokines noted in the authors' study may, as a result, indicate that physical activity is a beneficial factor in the reduction of inflammation among people with type 1 diabetes. On the other hand, the increased level of IL‐6 found among active men in the present research may be puzzling. In Belgian research, it was shown that resistance training decreased the level of IL‐6 in both sexes (Marynowski et al., 2021). However, in research carried out in Denmark, it was indicated that following exercise, the level of IL‐6 increases. Moreover, the increased level of this interleukin may have a beneficial effect on health because its heightened level in individuals with type 2 diabetes inhibits the production of pro‐inflammatory factors: TNF‐alpha and indirectly IL‐1. The results of these studies have been confirmed in additional cross‐sectional and longitudinal research. It has been shown that regular physical activity, also without losing weight, reduces systemic chronic inflammation (Karstoft & Pedersen, 2016). This is due to the fact that IL‐6 may have both pro‐inflammatory and anti‐inflammatory effects. It turns out that the IL‐6 produced by adipose tissue has pro‐inflammatory effects, while the one produced by myocytes demonstrates anti‐inflammatory influence. Physical activity in men stimulates myocytes to secrete this interleukin; hence, the increased level of IL‐6 in the studied men could have been due to increased secretion of this interleukin by myocytes (Galassetti et al., 2005; Karstoft & Pedersen, 2016). The elevated level of IL‐6 in the group of men declaring sufficient activity could also be related to the duration of the disease, which was declared as the longest in these individuals. Research conducted in the UK allowed to demonstrate that the level of Il‐6 during training changes, but the level of this adipokine correlated with the duration of the disease is not related to HbA_1c_ concentration or body fat (Turner et al., 2014).

In conclusion, the findings from this study allow to indicate that moderate level of physical activity may contribute to a reduction in the development of systemic low‐grade inflammation in patients with T1DM, and thus, may reduce the risk of CVD. This is evidenced by the fact that a detectable level of pro‐inflammatory IL‐8, that increases the risk of CVD was, found in individuals demonstrating low levels of physical activity. Such an observation is extremely relevant in light of the latest data indicating, that the prevalence of diabetes will increase in following years raised, which will further lead to an increase in costs of healthcare. Therefore, it is necessary not only to carry out further research to evaluate the influence of individual factors on the increased risk of diabetes and CVD in populations, but also to identify simple solutions that can effectively reduce this risk.

## 5 LIMITATIONS

The inconclusive results regarding the influence of physical activity on the level of pro‐inflammatory factors in the conducted research may be caused by the small sample size of the groups. The different level of producing pro‐inflammatory factors in response to fluctuations in hyperglycemia is associated with inter‐individual variation, and even variation in the response of a single patient. The level of IL‐6 is also influenced by the administration of insulin, which has an anti‐inflammatory effect (Rosa, Oliver, et al., 2011). Undoubtedly, the obtained results were also influenced by the fact that the separation of groups with low and sufficient levels of activity was carried out only on the basis of the IPAQ questionnaire. Therefore, it did not allow determination of the exercise types by the examined participants. What may also be puzzling is the fact that both women and men declaring a sufficient level of physical activity had a higher percentage of body fat than those from the reference groups. The observed trend may be the result of different diets in individual groups. It has been emphasized that regardless of the level of respondents' physical activity, obtaining a large amount of energy from lipids (Labayen et al., 2014) or eating a small number of meals a day contributes to an increase in body fat (Saneei et al., 2016). Therefore, research should be continued among a larger group of patients and concern a fixed training regimen.

## AUTHOR CONTRIBUTIONS

Małgorzata Kowal, Renata Woźniacka, and Anna Ścisłowska‐Czarnecka designed the experiment. Małgorzata Kowal and R.W. performed the experiment. Małgorzata Kowal and Renata Woźniacka did all thew calculations. Małgorzata Kowal wrote the first draft of this paper. Małgorzata Kowal, Renata Woźniacka, Anna Ścisłowska‐Czarnecka, and Wojciech Głodzik interpreted the results and contributed to later drafts, including the present manuscript. All authors were involved in data interpretation and critical revisions of the paper. All authors read and approved the final revised draft for publication.

## FUNDING INFORMATION

This study was funded by the University of Physical Education in Krakow (grant 229/BS/INB/2019).

## CONFLICT OF INTEREST STATEMENT

The authors declare that they have no conflict of interest.

## ETHICS STATEMENT

This study was approved by the Bioethics Committee (No. 196/KBL/012/2019), and the all respondents received written information on the course and purpose of the research and gave their written consent to participate in the trial.

## Data Availability

The data underlying this article are available in the article.
